# P53 An audit comparing current clinical practice against the British Society for Paediatric and Adolescent Rheumatology/the Arthritis and Musculoskeletal Alliance Standards of Care for children and young people with juvenile idiopathic arthritis in Grimsby since 2015

**DOI:** 10.1093/rap/rkac067.053

**Published:** 2022-09-28

**Authors:** Emily Pawley, Anne-Marie McMahon, Pauline Adiotomre, Helen Lee

**Affiliations:** Diana Princess of Wales, Grimsby, United Kingdom; Sheffield Children's Hospital, Sheffield, United Kingdom; Diana Princess of Wales, Grimsby, United Kingdom; Diana Princess of Wales, Grimsby, United Kingdom

## Abstract

**Introduction/Background:**

Our aim was to describe current clinical practice against the British Society for Paediatric and Adolescent Rheumatology (BSPAR)/Arthritis and Musculoskeletal Alliance (ARMA) standards of care (SOC) for children and young people (CYP) with juvenile idiopathic arthritis (JIA) in Grimsby since 2015, in 3 set domains.

The 3 criteria were: mean time from referral to an appointment in a specialist paediatric rheumatology centre (PRH03); mean time for children who need an intra articular steroid injection to have it, calculated from the date of decision to treat (PRH04); mean time from diagnosis of JIA to first uveitis screening (PRH05)

**Description/Method:**

Briefly, JIA is a heterogenous group of conditions, of unknown cause, with symptoms lasting over 6 weeks, with an onset before age 16 years. Typically, there is joint pain and inflammation. There are 6 types of JIA: oligoarticular, polyarticular, systemic, psoriatic, enthesitis-related, and undifferentiated.

For some background on our services, there are monthly paediatric rheumatology outreach clinics, held between Sheffield Children’s Hospital (tertiary centre) and Diana Princess of Wales, Grimsby. Patients with both inflammatory and non-inflammatory conditions are seen, with currently 33 patients under this outreach service. There has not been recent research into whether the standards of care, made in 2009, are actually being met, and if not then by how much. Therefore, there was a clear gap in research, with a potential for eye-opening results.

Of these 33 patients, 13 were diagnosed with JIA (between 2015 and 2022) and therefore were used for the audit. Data was obtained and analysed, with the percentages meeting each standard of care as follows:

- Time from referral to an appointment in Sheffield: 30.7% (ranging from 3 days to 73 days)

- Time from decision to treat to having first steroid injection: 84.6% (ranging from 0 days to 64 days)

- Time from diagnosis to first uveitis screening: 46.2% (ranging from 3 days to 223 days)

From this, we can conclude that the least met standard of care was time from referral to an appointment in Sheffield. However, none of these results are 100%, therefore suggesting room for improvement is required across all domains.

**Discussion/Results:**

The next question to assess is why these percentages are not 100%, and what the multiple barriers are to reaching the targets. In addition, how we can overcome them to improve these numbers as practitioners.

There have been other larger-scale studies previously to also demonstrate the lack of meeting targets. Even with the introduction of the SOCs, in addition to an increasing awareness of the negative impact of delay in access to paediatric rheumatology care on disease outcomes in JIA patients, a multi-site UK audit done in 2013 against key SOCs showed significant variation in time to access specialist care and service delivery. It therefore suggests that the results from our research might well be similar currently across the UK, not just in Grimsby/Sheffield. Studies need to be done across the UK to explore this.

These SOCs were published in 2009 and have not been reviewed/renewed since. In addition, there are currently no incentives for the hospital to meet the criteria, with the standards only laid out as ‘guidelines’, which could well in turn be having an impact on the lower percentages that we have seen in the results. Hopefully, supported by the evidence we have from the study that change is needed, this can be reviewed, so that we can improve the care we give to our patients.

Education on why it is vital that we meet this criteria is also important, for both practitioners and the general public. There might well be insufficient training for juniors on JIA, including spotting it for diagnosis and the referral pathway once diagnosed. This could be a reason for it taking a longer period of time for the patients to see a specialist in the tertiary centre (Sheffield).

Any ideas or views on how these results could be improved are welcome.

**Key learning points/Conclusion:**

It has been a new learning experience coming across and attending the Grimsby/Sheffield outreach clinics, including learning simply that they exist. I have been able to see in person how they are structured, and the importance of them. These clinics also allowed me to meet some of the patients who were included in our audit project, which was nice. Furthermore, it has been a rewarding experience auditing data that is now being discussed amongst the Sheffield/Grimsby teams, with a strong aim to improve clinical practice.

In terms of doing the audit - I am not yet very experienced with research projects, so it was good to recap basic research skills, and the principles of auditing. This included reading around the topic and referencing any useful publications to obtain some background, prior to then delving into our set research. I’ve also been reminded of the usefulness of tables – a simple tool, I discovered, that can effectively demonstrate any trends.

From a perspective of a junior doctor … the topic of the audit has also allowed me to recap and further my knowledge around JIA, from diagnosing it to learning the specific criteria that are in place for managing JIA. Interestingly, as I previously mentioned in terms of perhaps a lack of education available on the standards of care, I myself had not come across any set SOCs prior to the audit. It was useful and eye-opening to see that BSPAR published set criteria that should be met for JIA patients, to meet their various needs.

